# Supplementary material for: NK Cell Subset Redistribution and Antibody Dependent Activation after Ebola Vaccination in Africans
Source: Vaccines (Basel). 2022 May 31;10(6):884. doi: 10.3390/vaccines10060884 (PMC9230153; doi:10.3390/vaccines10060884)
Supplement: Supplementary file 1 [file vaccines-10-00884-s001.zip › vaccines-1684456-supplementary.pdf]

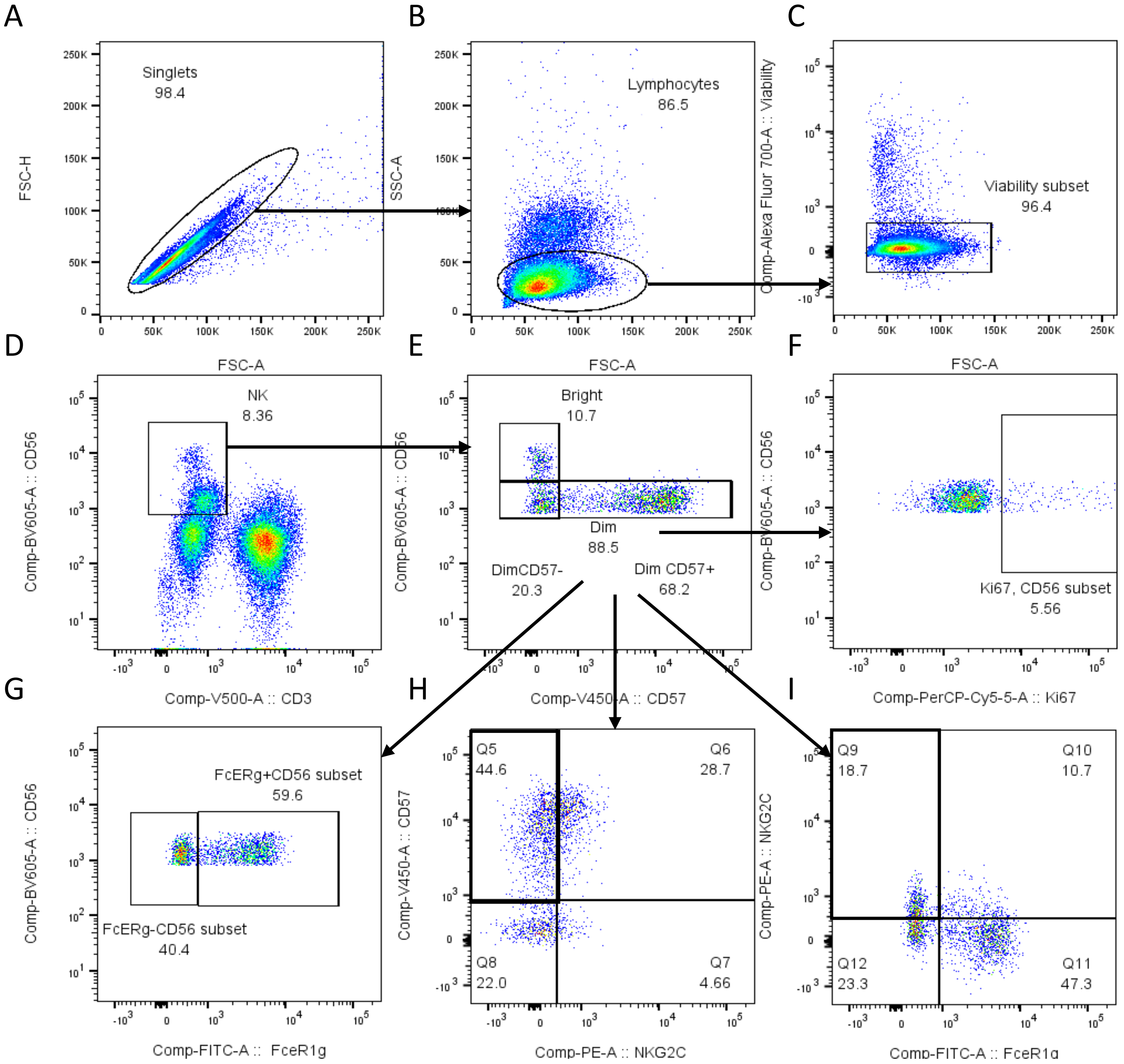

Figure S1. Gating strategy for NK cell subsets measured ex-vivo

For ex-vivo flow cytometric analysis, PBMC were first gated on singlet events (**a**) followed by lymphocytes (**b**) and viable lymphocytes (**c**). NK cells were defined as $CD3^{-}CD56^{+}$ (**d**) and gated into $CD56^{bright}$ or $CD56^{dim}$ subpopulations according to CD57 (**e**), Ki67 (**f**) and adaptive NK cell subsets defined by FcεR1γ alone (**g**) and CD57/NKG2C (**h**) or NKG2C/FcεR1γ expression (**i**).

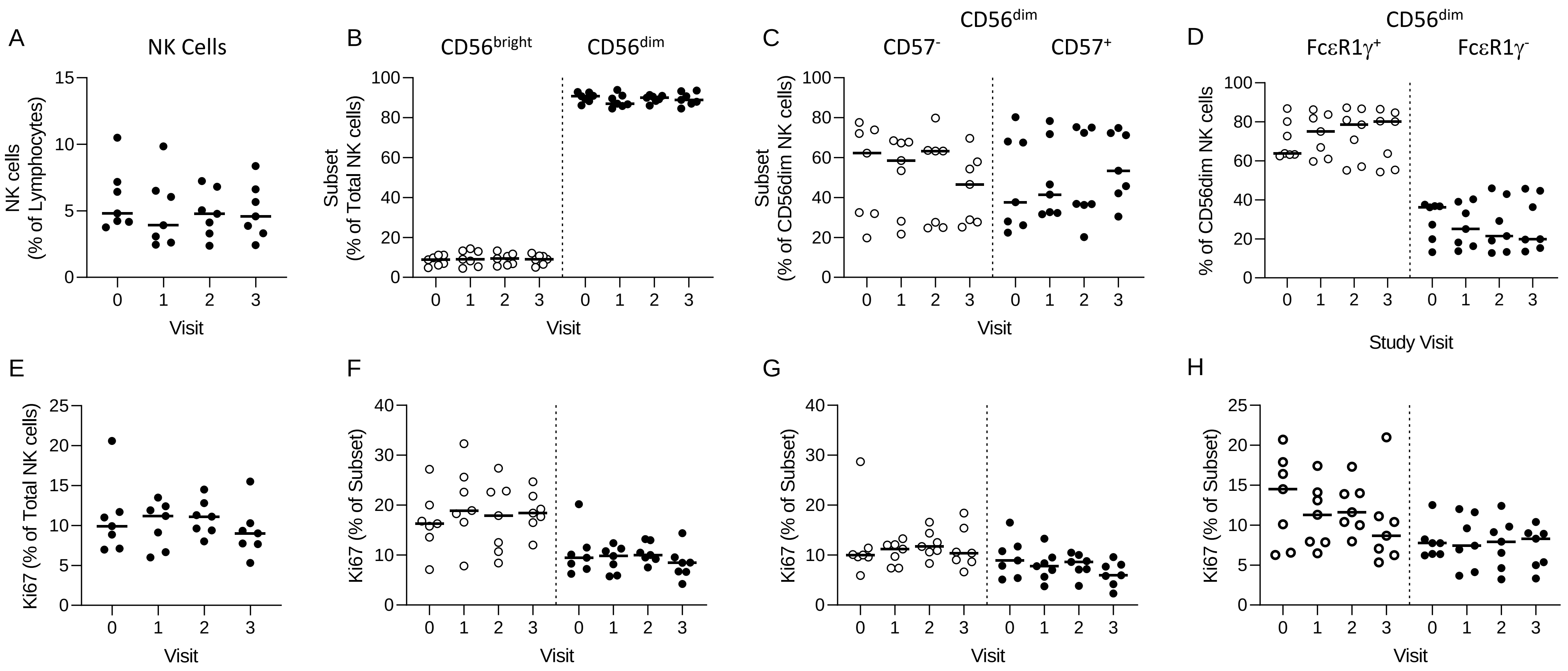

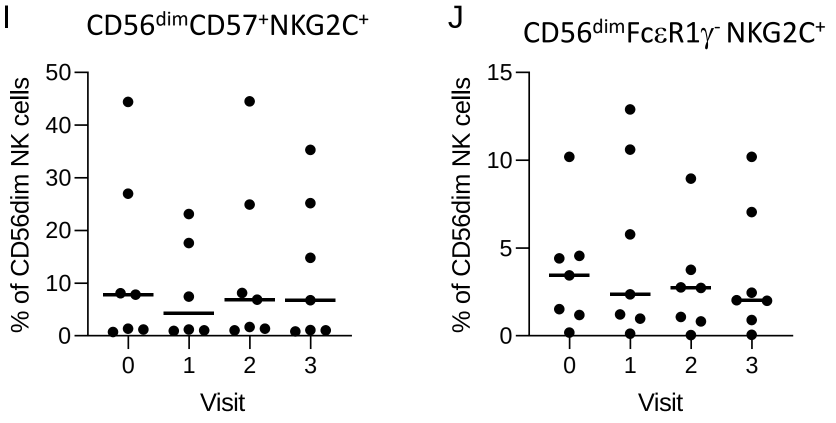

Figure S2. *Ex vivo* analysis of NK cell subsets and activation markers in placebo controls.

Frequencies of NK cells and subsets at Visit 0 (baseline), Visit 1 (post-vaccine dose 1), Visit 2 (21 days post vaccine dose 2) and Visit 3 (180 days post-vaccine dose 2) (**a**) NK cells (**b**) $CD56^{bright}$ and $CD56^{dim}$ (**c**) $CD56^{dim}CD57^{-}$ and $CD57^{+}$ and (**d**) $CD56^{dim}Fc\varepsilon R1\gamma^{+}$ and $Fc\varepsilon R1\gamma^{-}$ **(e)** Ki67 expression within NK cells and within (**f**) $CD56^{bright}$ and $CD56^{dim}$ (**g**) $CD56^{dim}CD57^{-}$ and $CD57^{+}$ and (**h**) $CD56^{dim}Fc\varepsilon R1\gamma^{+}$ and $Fc\varepsilon R1\gamma^{-}$ NK cell subsets (**i**) Frequencies of $CD57^{+}NKG2C^{+}$ and (**j**) $Fc\varepsilon R1\gamma^{-}NKG2C^{+}$ subsets within $CD56^{dim}$ NK cells across the vaccination time course. Placebo control study participants corresponding to vaccination groups 1 and 2 were pooled for analysis (n=7). Paired comparisons between time points were made using Wilcoxon signed-rank test.

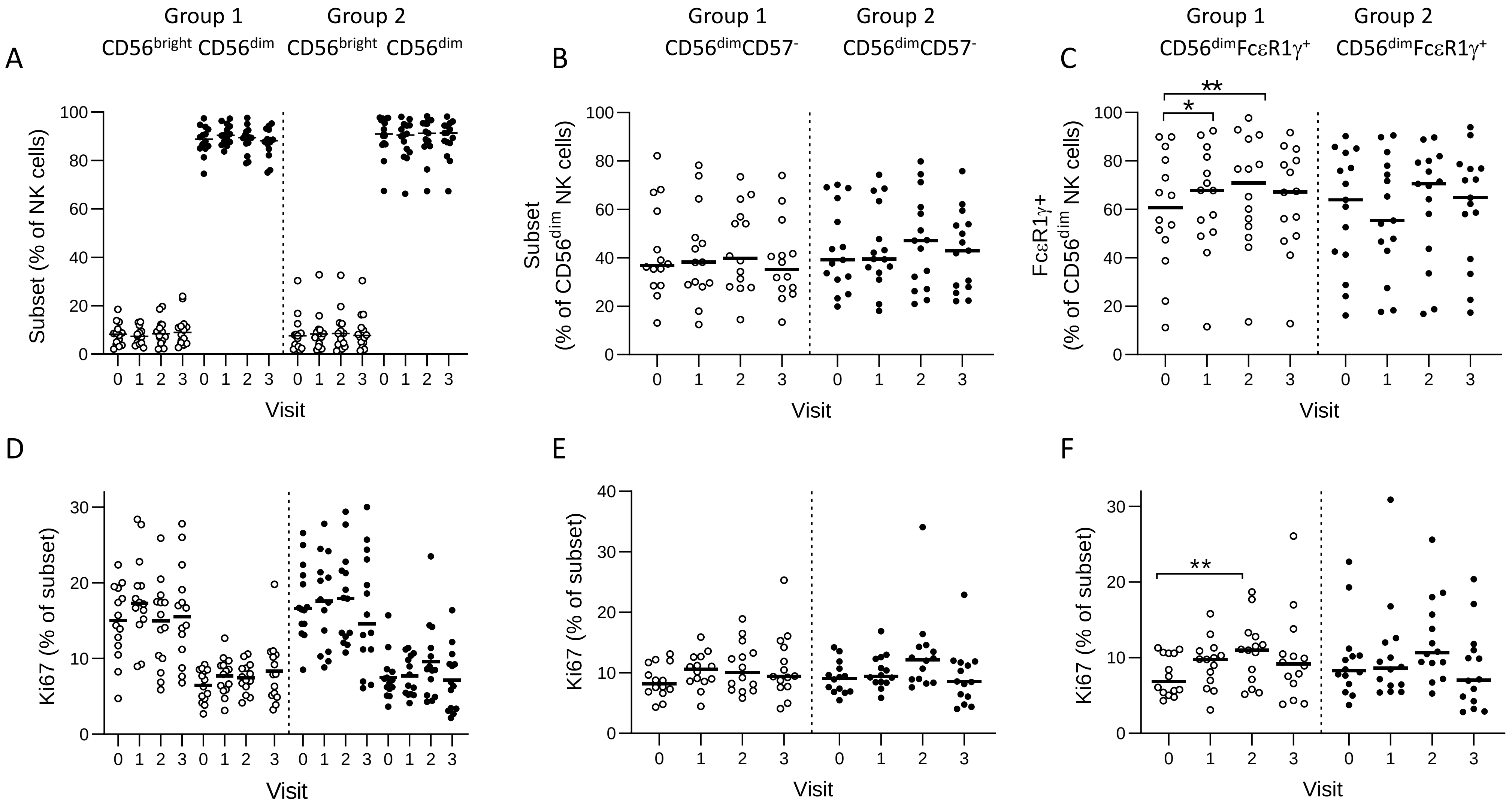

Figure S3. Impact of dosing interval on NK cell subset distribution *ex vivo*

Ex vivo flow cytometric data was analysed in actively vaccinated individuals in Group 1 (dose 2 = 28 days post dose 1; n=14) and Group 2 (dose 2 = 56 days post dose 1; n=15). Overall frequencies of $CD56^{bright}$ and $CD56^{dim}$ NK cells (**a**) and CD57 (**b**) or FcεR1γ (**c**) defined subsets and the frequency of Ki67 within each subset (**d-f**) is shown for both groups. Comparisons between paired data points within each group were made using Wilcoxon signed-rank test. * $p<0.05$, ** $p<0.01$.

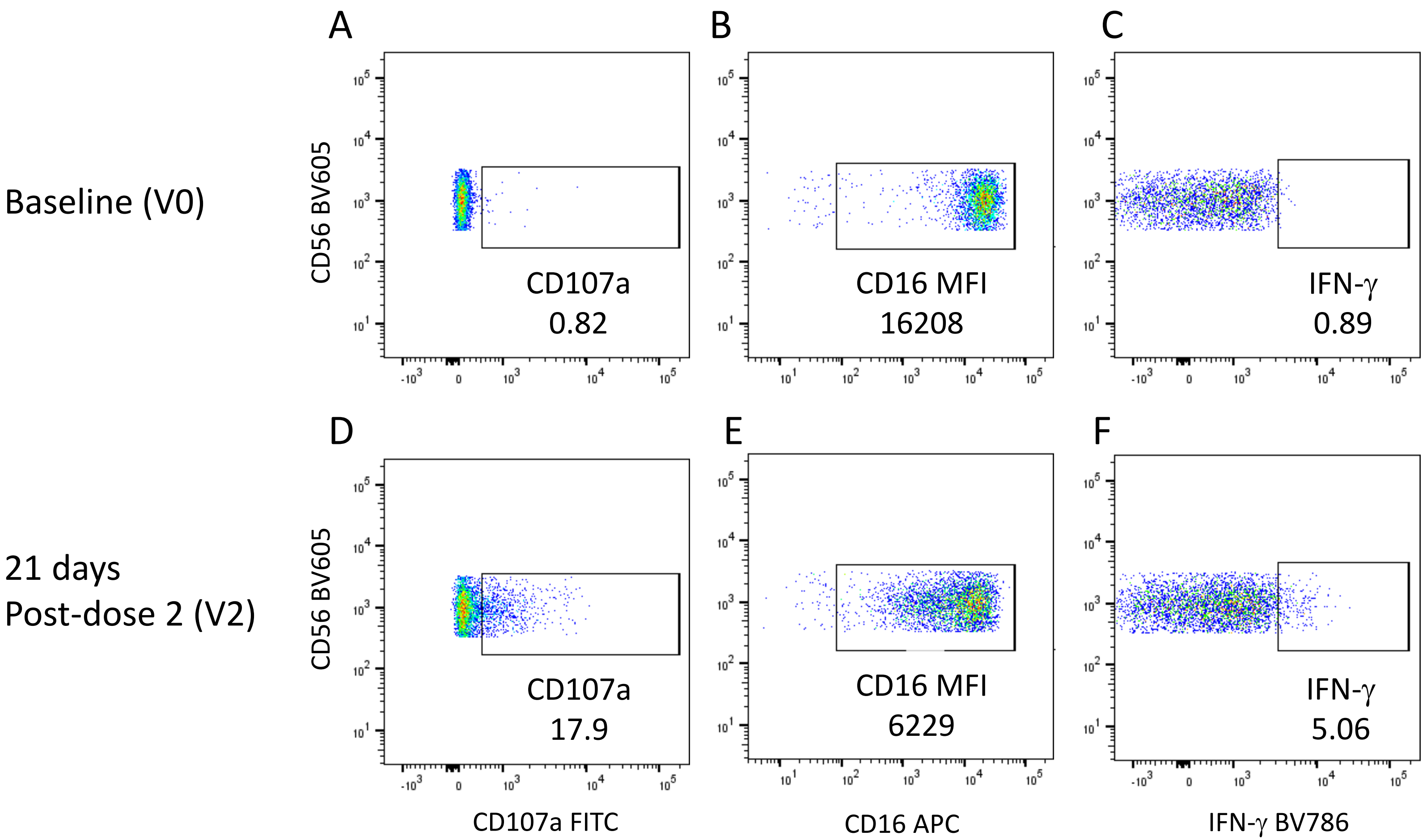

Figure S4. Gating strategy for in-vitro ADNKA assays

ADNKA responses were analysed after culture with recombinant Ebola glycoprotein and pre- or post-vaccination sera. Pseudocolour plots are shown for CD107a (**a, d**), CD16 (**b, e**) and IFN-γ (**c, f**) expression within gated CD56^{dim} NK cells from a representative donor after culture with baseline (V0) serum (**a-c**) or post-dose 2 (V2) (**d-f**) serum.

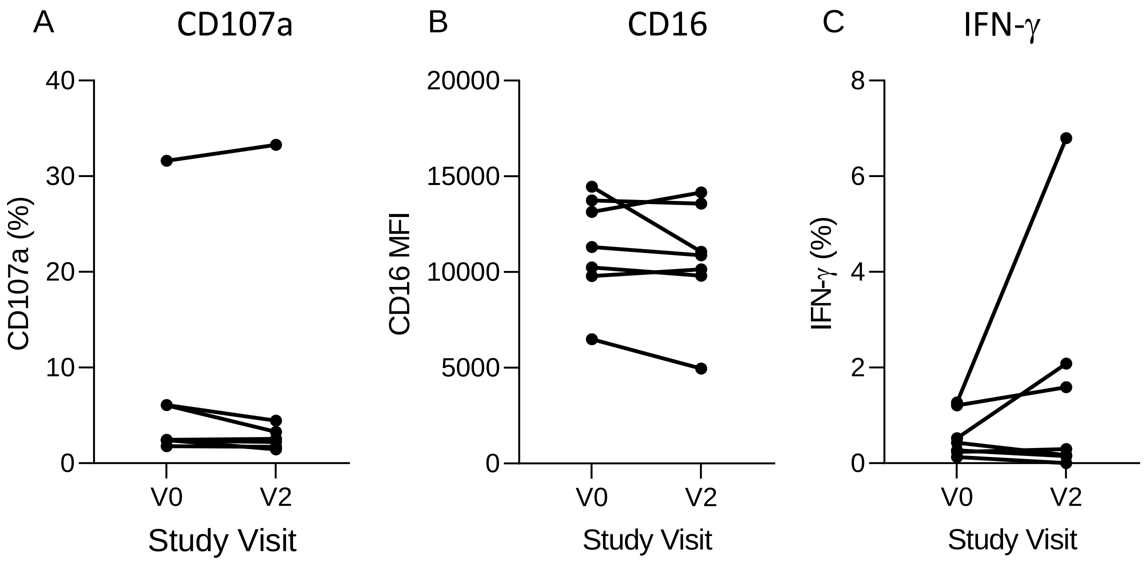

Figure S5. Antibody-dependent NK cell responses to immobilized EBOV GP in placebo controls.
CD107a (**a**), CD16 MFI (**b**) and IFN-γ (**c**) expression within gated $CD56^{dim}$ NK cells from a standard PBMC readout (from a single unvaccinated donor) cultured with serum samples from individual trial donors collected pre-vaccination (Visit 0) and 21 days post-dose 2 (Visit 2). Data are presented for combined vaccination groups and plots show individual data points before after vaccination (**a-c**).

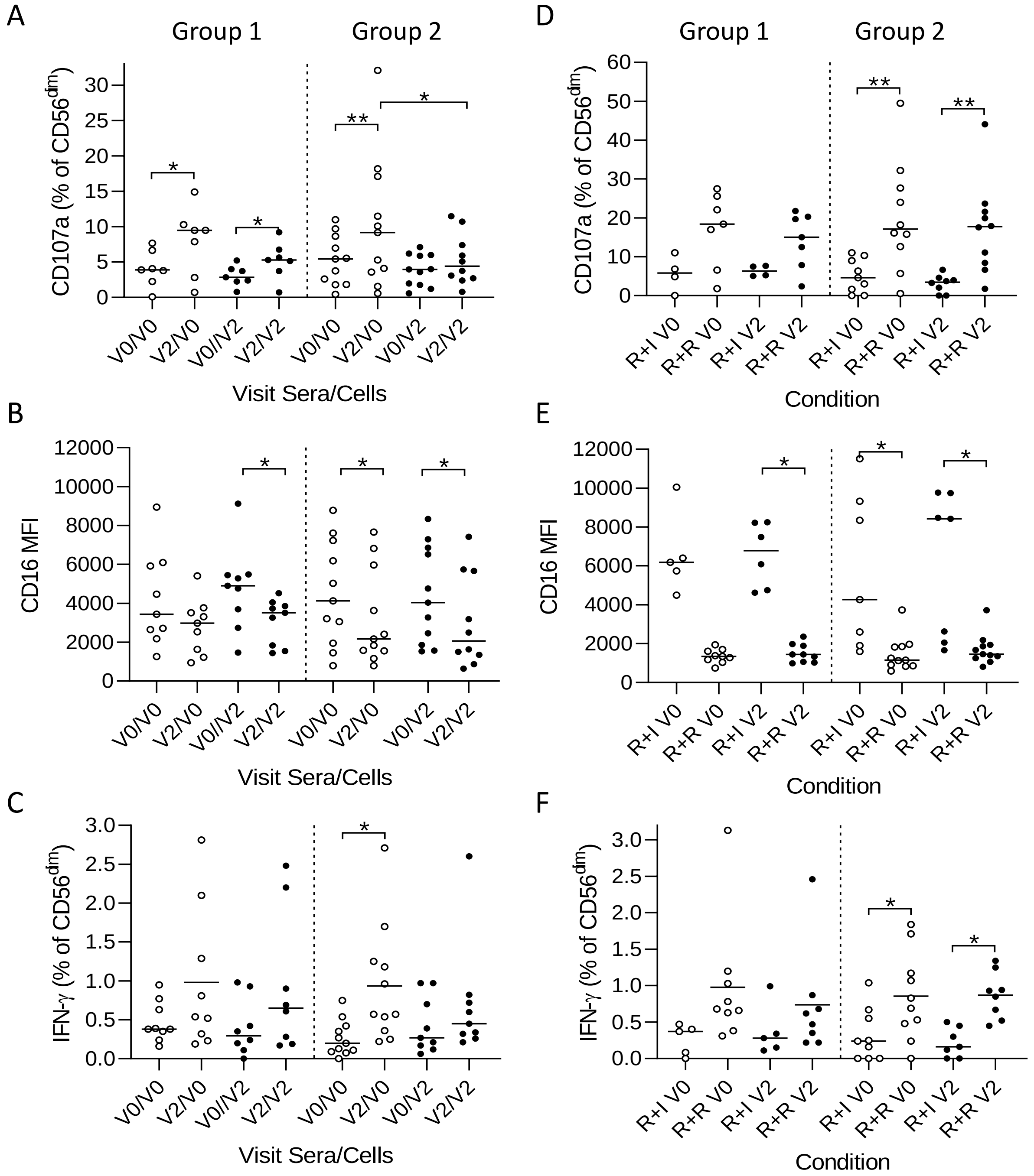

Figure S6. The Impact of dosing interval on ADNKA responses of autologous NK cells

ADNKA responses were analysed in actively vaccinated individuals in Group 1 (dose 2 = 28 days post dose 1; n=8) and Group 2 (dose 2 = 56 days post dose 1; n=10). PBMC collected pre-vaccination (V0) and 21 days post-dose 2 (V2) were cultured with EBOV GP in the presence of autologous serum from the same visit or with pooled serum from Visit 2 (V2p) (**a-c**). Positive and negative control stimuli were Raji B cells + Rituximab™ (R+R) or isotype control antibody (R+I), respectively (**d-f**). Responses were analysed by flow cytometry for (**a, d**) CD107a, (**b, e**), CD16 mean fluorescence intensity (MFI) and (**c, f**) IFN-γ expression. Individual data points are shown for 18 participants tested in this assay with a line representing median values for all tested donors. Comparisons between visits were performed using one-way ANOVA mixed effects analysis with Geisser-Greenhouse correction. *p<0.05, ** p<0.01.
